# CGHPRO – A comprehensive data analysis tool for array CGH

**DOI:** 10.1186/1471-2105-6-85

**Published:** 2005-04-05

**Authors:** Wei Chen, Fikret Erdogan, H-Hilger Ropers, Steffen Lenzner, Reinhard Ullmann

**Affiliations:** 1 Max-Planck Institute for Molecular Genetics, Ihnestrasse 73, 14195 Berlin, Germany

## Abstract

**Background:**

Array CGH (Comparative Genomic Hybridisation) is a molecular cytogenetic technique for the genome wide detection of chromosomal imbalances. It is based on the co-hybridisation of differentially labelled test and reference DNA onto arrays of genomic BAC clones, cDNAs or oligonucleotides, and after correction for various intervening variables, loss or gain in the test DNA can be indicated from spots showing aberrant signal intensity ratios.

Now that this technique is no longer confined to highly specialized laboratories and is entering the realm of clinical application, there is a need for a user-friendly software package that facilitates estimates of DNA dosage from raw signal intensities obtained by array CGH experiments, and which does not depend on a sophisticated computational environment.

**Results:**

We have developed a user-friendly and versatile tool for the normalization, visualization, breakpoint detection and comparative analysis of array-CGH data. CGHPRO is a stand-alone JAVA application that guides the user through the whole process of data analysis. The import option for image analysis data covers several data formats, but users can also customize their own data formats. Several graphical representation tools assist in the selection of the appropriate normalization method. Intensity ratios of each clone can be plotted in a size-dependent manner along the chromosome ideograms. The interactive graphical interface offers the chance to explore the characteristics of each clone, such as the involvement of the clones sequence in segmental duplications. Circular Binary Segmentation and unsupervised Hidden Markov Model algorithms facilitate objective detection of chromosomal breakpoints. The storage of all essential data in a back-end database allows the simultaneously comparative analysis of different cases. The various display options facilitate also the definition of shortest regions of overlap and simplify the identification of odd clones.

**Conclusion:**

CGHPRO is a comprehensive and easy-to-use data analysis tool for array CGH. Since all of its features are available offline, CGHPRO may be especially suitable in situations where protection of sensitive patient data is an issue. It is distributed under GNU GPL licence and runs on Linux and Windows.

## Background

DNA sequence copy number changes have shown to play an important role in the aetiology of cancer and congenital disorders. Comparative Genomic Hybridization (CGH) is a molecular cytogenetic method for the detection of chromosomal imbalances [1], which does not depend on the availability of chromosome spreads and is not confined to the analysis of growing cells. Unfortunately, conventional chromosomal CGH has a low resolution. Recently, this drawback has been overcome by the introduction of array CGH. Here differentially labelled test and reference DNA are co-hybridized onto microarrays of several thousand evenly spaced DNA clones or oligonucleotides representing specific regions of the human genome[2,3]. The resolution of this technique depends on the number of different DNA spots printed on the glass slides and on the size of the DNA clones used. Currently most available CGH arrays allow the reliable detection of deletions and duplications if they are larger than 1 Mb. However, with arrays containing more densely spaced BAC (Bacterial Artificial Chromosomes) clones containing on average 150 kb of human DNA, much higher resolutions can be achieved [4]. Up to now, array CGH has been predominately used in highly specialized laboratories, and most of the data analysis programs currently available are not able to process the output of array CGH experiments in an easy and comprehensive way. For example, the two R packages, aCGH and DNAcopy, can identify copy number transitions on chromosomes by Unsupervised Hidden Markov Model [5] and Circular Binary Segmentation [6], but the application of these tools requires basic programming skills in R language. CGH-Plotter is a MATLAB toolbox with a graphic user interface. It detects the regions of amplifications and deletions using k-means clustering and dynamic programming. However, like aCGH and DNAcopy, CGH-Plotter can only be used to analyse already normalized array data in a specific format [7]. In addition, these programs output display the results in a non-interactive plot. As a visualization tool for array CGH, SeeGH can display the data in a user friendly interface[8]. It allows users to explore the results in a conventional karyotype diagram with annotation. However, without the essential statistical methods for characterizing the genomic profile, seeGH is rather a visualization than an analysis tool for array CGH.

Here we present a comprehensive data analysis software for array CGH. The program combines analysis and visualization of array CGH data. Furthermore it supports comparative analysis of complete study groups based on absolute and relative frequencies of aberrations.

## Implementation

### Software design and information sources

CGHPRO was programmed in Java and MySQL was used as the back-enddatabase. The decision to use Java and MySQL was based on their public availability, their platform independence and the fact that MySQL can handle large data files with high throughput. Two "R" packages from Bioconductor[9], DNAcopy and aCGH, were implemented in our software, which enable a platform-independent characterization of genomic profiles. Up to now, CGHPRO has been tested in a Linux and a Windows 2000 environment.

To date, CGHPRO allows the import of result files from GenepixPro5.0, Agilent and Imagene, but users can also customize the program to support their own data format. All essential data required to meet future standards of "minimal information about an array CGH experiment" can be stored in the database. Such standards have already been defined for gene expression analysis [10], but are still lacking for array CGH.

The genome annotation that has been integrated into the current version of CGHPRO is based on the UCSC Genome Browser [11], but users can choose different versions to meet their own needs.

The example chosen for demonstration of this software is a male versus female co-hybridization onto a 14000 BAC array, comprising a genome wide 1 Mb resolution BAC array (clones kindly provided by Nigel Carter, Wellcome Trust Sanger Centre, [12]) and the tiling path of nine chromosomes from the Human "32 k" BAC Re-Array set, a series of overlapping BAC clones obtained from BACPAC Resources Centre at Children's Hospital Oakland Research Institute [13,14]. Detailed protocols for the generation of the arrays and the hybridization are available at our website [15].

The dataset for the comparative analysis was generated artificially.

## Results and discussion

### Database design

In CGHPRO a back-end database using MySQL has been implemented. The database stores the description of each analyzed chip (glass slide) as an entry in the table 'analyzedChips'. This description includes all essential information about the experimental and data analysis procedure, e.g. the number of spots that have been excluded and the normalization algorithm applied. A separate table named according to the Chip ID saves the original data from the image analysis software as well as the results from data analysis. A table called 'clonePosition' is used to store the mapping information for each clone. The information comprises data that might influence the reliability of the clone's hybridization characteristics, such as content of repetitive sequences and most importantly, its involvement in segmental duplications, which can be visualized by a colour code, as discussed below. In order to be able to use this feature properly, users with cDNA- or oligo-arrays have to adjust the chromosome positions of their probes. Instead of the actual chromosome position, one should define the chromosomal region, which the probe represents.

### Data input

CGHPRO allows the import of output files from several image analysis software packages, as listed above. Essential data are extracted and spots flagged as "poor" by the image analysis software are excluded automatically. Mapping information and related annotations for each clone are fetched from the back-end database.

Mapping information for each clone, based on a specific version of UCSC Genome Browser, has to be provided by the user. For this purpose, a tab-delimited file has to be loaded into the back-end database, which must include six fields for each clone, the unique identifier, the respective chromosome, the positions of the first and last base pair, the source of the clone, and the user-specified comments of the clone. For the complete tiling path, as distributed by the BACPAC Resources Centre, the mapping information based on the April 2003 assembly of UCSC Genome Browser comes along with the software.

The way this information is acquired differs from other recently published programs like ArrayCGHbase [16] or CAP [17], both of which provide these data by directly accessing the respective genome browser. This may be an advantage when looking for the most recent update, but it may pose problems for diagnostic and related applications, where patient confidentiality is important and precludes online data analysis. Offline analysis also speeds up the process, as it is not dependent on server capacities or transfer rates.

### Graphical analysis of hybridization characteristics

CGHPRO provides a variety of graphical data representation tools to visualize the data before and after normalization. Scatterplots allow for estimates of the noise within a given data set. Spatial dependency of log ratios can be detected with Boxplots for all different subgrids. MAplots are implemented to detect intensity-dependent effects of the log ratios distribution [18], and the distributions of signal intensity ratios are displayed as histograms. This feature is supplemented by QQ plots, which demonstrate to what extent the ratio follows the normal distribution.

Visual inspection enables identification of clones that should be excluded from further analysis, and at the same time it provides a basis for choosing the appropriate method of normalization.

### Data normalization

The goal of normalization is to remove any systematic bias in the measured fluorescence intensities. Such systematic bias can originate from different labelling efficiencies of the used fluorochromes, different scanning parameters, spatial effects and/or other effects. In CGHPRO there are four options to remove these biases: Global Median, LOWESS (Locally Weighted Regression and Smoothing Scatterplots) [19], Subgrid Median and Subgrid LOWESS. Global methods assume that the bias is constant across the whole chip. Using Global Median, the Median value of log2 ratios is chosen as the correcting value. LOWESS function is especially useful for removing intensity-dependent biases. In order to reduce the influence of spatial effects, as may be detected by Boxplots, the above two methods can be applied to every subgrid separately. According to our experience, at least in our settings, the median subgrid works best for the normalization of array CGH data.

If one clone is spotted more than once on the chip, the replicate spots are automatically identified by their common ID. After normalization, the normalized ratios for the replicates are averaged and the standard deviations are calculated. This average ratio will later be used to represent the ratio of each clone. In subsequent analysis, users can set a threshold based on the number of replicas and standard deviation, such that clones exhibiting inconsistent results can be excluded.

### Characterization of genomic profiles

The eventual goal of array CGH is the characterization of the individual genomic profile. Up to now, the common method is to use fixed thresholds, which should be dependent on the variability of the data. CGHPRO allows users to set the threshold either directly, or smooth the data first and then set a threshold based on the smoothed results. For smoothing, CGHPRO provides two options. When using moving average, which is applied to each chromosome separately, a window of adjustable size moves along the clones, which are ordered according to their base pair positions on the chromosome. The smoothed ratio of the clone at a window's centre will be the average ratio of the clones within the window.

The second smoothing strategy is to segment the clones, which are ordered along the chromosome, into sets with equal copy numbers. Then the data can be smoothed via averaging within the sets.

CGHPRO includes two methods for the segmentation of chromosomes into regions with identical copy numbers, namely 'Unsupervised Hidden Markov Partition' created by Jane Fridlyand [5] and 'Circular Binary Segmentation' first published by Adam Olshen [6]. We have implemented the two methods by linking the two R packages, aCGH and DNAcopy, to our program. Based on the smoothed ratios generated by one of these two algorithms, we have introduced the Median Absolute Deviation (MAD) as an objective measurement of data scattering.

### Data display

#### Genomic display

**Figure 1:**
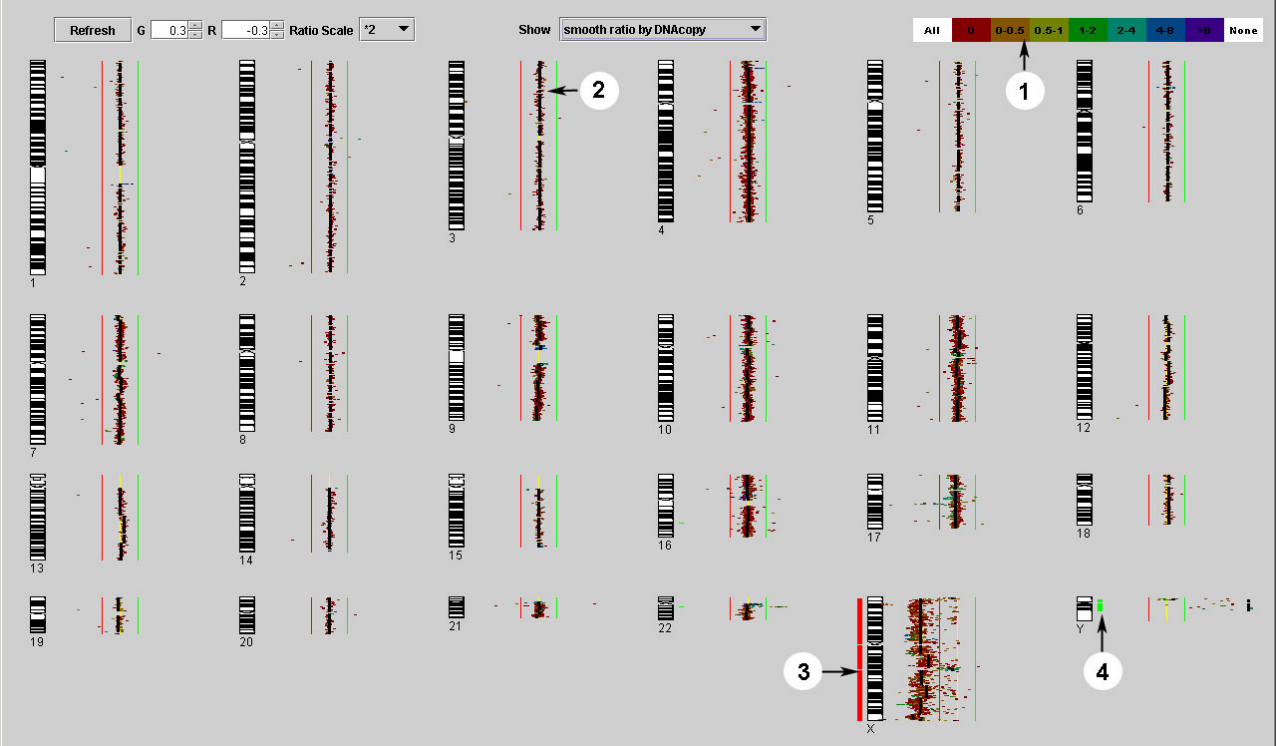
**Genome Display exemplified by a male versus female hybridization on a 14000 BAC DNA array: **Circles 1–4: (1) Colour coding table indicating the involvement of clones in segmental duplication (2) Black line representing the smoothed ratios calculated by DNAcopy (3) and (4) red and green bars to the left and right side of the ideogram highlighting regions of losses and gains, respectively.

The graphical interface of CGHPRO allows to explore the results in an interactive interface (Figure 1). In the Genome Display, the window consists of 24 sub-panels, each containing one chromosome. In each sub-panel, the ratios of clones are plotted in a size-dependent manner along the ideogram. As described below, several display parameters can be modified.

In each sub-panel, there are three lines along each chromosome. The yellow line represents a log ratio of zero; the individually adaptable green and red lines mark the negative and positive log ratios, respectively. The smoothed log ratios calculated by moving average, DNAcopy or aCGH can be chosen to be displayed as a black line called Smooth Line. Optionally, the original data can be blanked out.

Each clone is colour-coded according to its involvement in segmental duplications, as defined by the following formula: (Σ Length of Duplication * Copy Number)/ Length of Clone. Based on the factors determined this way, the clones are grouped into seven classes that can be looked up separately by clicking on the button with the corresponding colour in the top right corner.

Segmental duplications, which comprise ~5% of the human genome, are copies of genomic DNA with >90% sequence identity that range in size from 1 to >200 kb and are present in at least two locations in the human genome [20]. Highlighting segmental duplications is useful for the recognition of clones that may show misleading ratio scores [21]. Moreover, this feature also allows to relate chromosomal rearrangements to duplicated genomic regions. It has already been shown that segmental duplications increases the chances of non-allelic homologous recombination and that genomic regions flanked by these duplications are particularly prone to rearrangements [22]. Clicking on each sub panel will open a separate window and allow zooming on a specific chromosome.

#### Chromosome display

**Figure 2:**
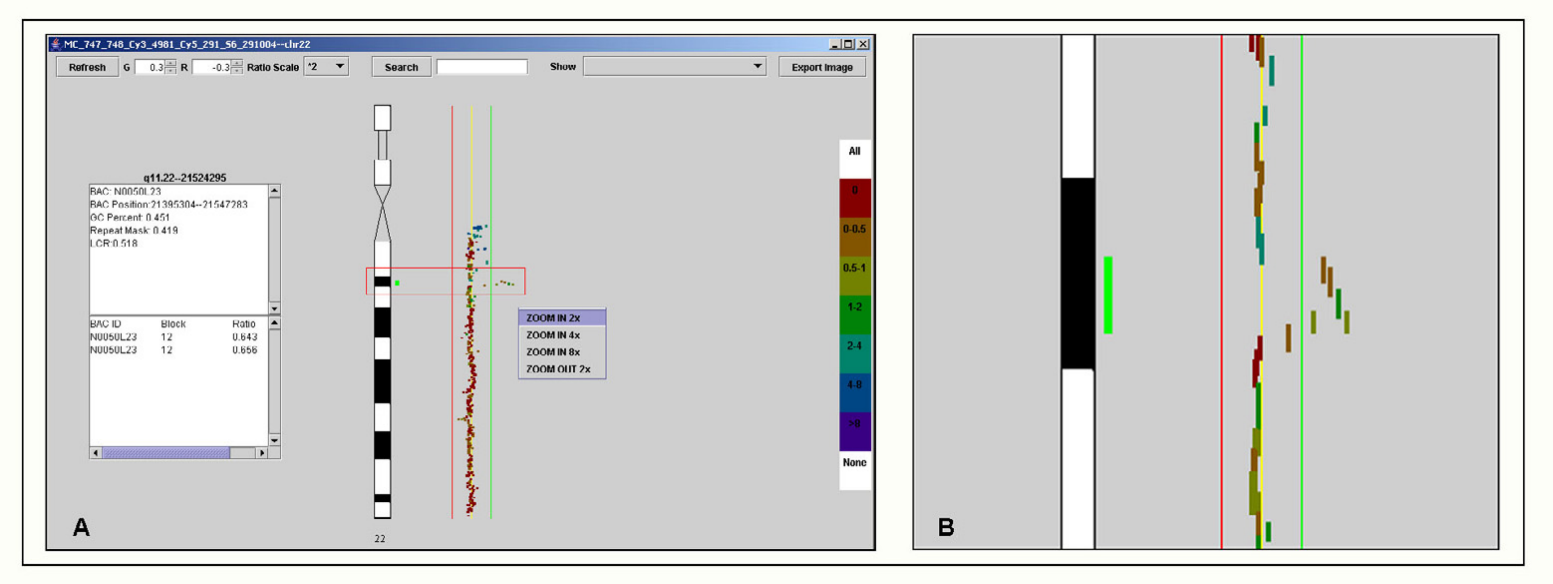
**Chromosome Display: **(A) Detection of a copy number polymorphism, which encompass about 300 kb. (B) Zoom-in view of the relevant region (red rectangle in (A)).

Chromosome Display provides a detailed view of the selected chromosome (Figure 2). In addition to the features provided by the Genome Display, the Chromosome Display supports the search for clones, zooming in or out, as well as the export of images. Upon clicking on a clone, information on its exact localization, contents of simple repeats, its involvement in segmental duplications, as well as information on number, position and ratio of the present replicas will be displayed in a text box. A key feature added to the Chromosome Display is a right-click mouse event, which will open a pop-up menu, offering several zoom options. Finally, Chromosome Display can be exported as an image file in Portable Network Graphics (png) format.

### Comparative analysis of different chips

**Figure 3:**
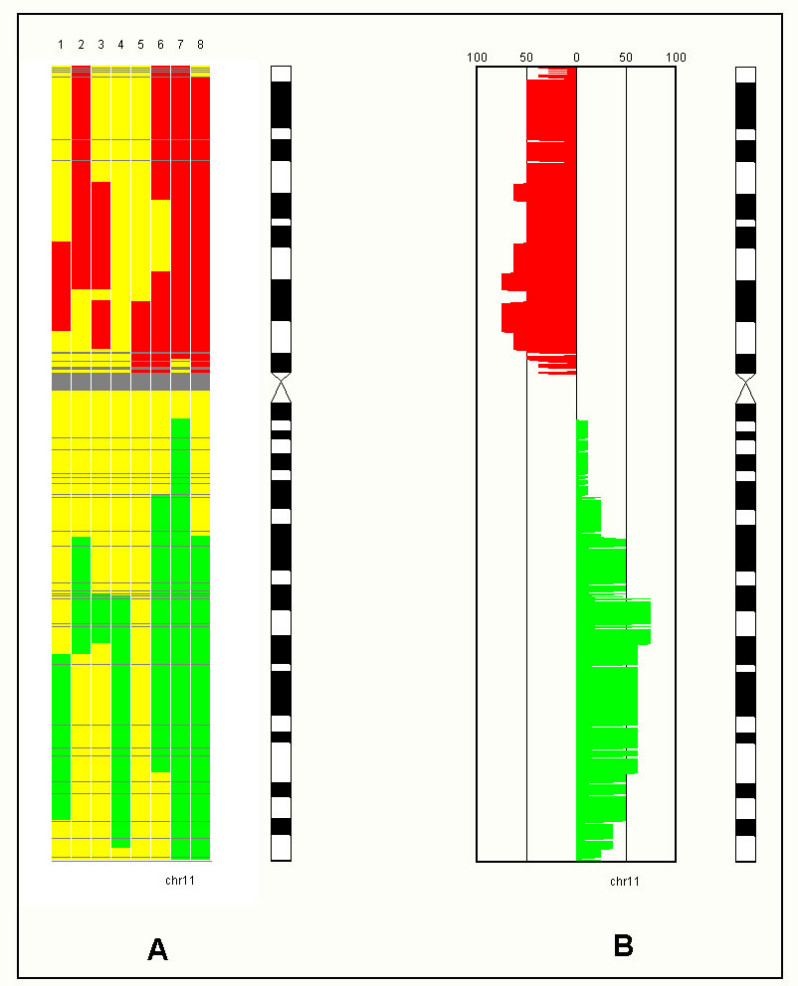
**Comparative Analysis: **CGHPRO supports the visualization of absolute (A) and relative (B) frequencies of chromosomal aberrations in a series of cases. Results can be displayed simultaneously for all or for single chromosomes, as shown here for chromosome 11.

Once stored in the database, all entries can be used for comparative analysis at the genomic, chromosomal and clone-by-clone level. Genomic View is especially suitable for the summarizing display of chromosomal aberrations in a series of cases. In this mode, the absolute frequencies of aberrations within a study group are displayed alongside the chromosome ideograms ordered in a 6 × 4 grid. Upon clicking on the chromosomes of interest in the list located at the left side of the screen, the program switches to the Chromosome View and zooms in the respective chromosome. In addition to the absolute frequencies of aberrations, the relative frequencies can also be shown, which makes it easier to compare study groups of different size (Figure 3). For detailed analysis, the clone-by-clone view can be used. This mode supports "mouse over functionality", which displays further clone information in the bottom text field. Additionally the clone-by-clone matrix paves the way for the implementation of further algorithms such as hierarchical clustering.

As in all other views, balanced regions are indicated in yellow, while deleted and gained regions are shown in red and green, respectively. The simultaneous display of results from several experiments can assist in the definition of shortest regions of overlap, can help to reveal patterns of chromosomal aberrations, and can facilitate the recognition of odd clones.

## Conclusion

CGHPRO represents a comprehensive and easy-to-use data analysis tool for array CGH. The software features the test of hybridization quality, normalization and visualization, as well as interactive data exploration and the comparative analysis of complete study groups. By providing all features offline, CGHPRO can be especially suitable in situations where protection of sensitive patient data is an issue. CGHPRO is written in Java and requires MySQL and R. The program runs on Linux and Windows operating systems.

It is freely available for use under the terms of the GNU General Public Licences (GPL) at the project's homepage. The open design of CGHPRO allows the easy adaptation to specific needs and the future incorporation of new features.

## Availability and requirements

Project name: CGHPRO

Project home page: 

Operating system: Linux and Windows 2000

Programming language: Java, SQL, R

Other requirements: Java 1.4 or higher, MySQL database and R

License: GNU General Public License

Any restriction to use by non-academics: Contact authors

## Authors' contributions

WC was the principal programmer of the CGHPRO software. FE has performed the hybridization experiment and tested the program, HHR and SL contributed ideas for display and analyzing features and RU was responsible for defining the practical requirement catalog and contributed to manuscript preparation.
